# Cut-Off Analysis of CTC Change under Systemic Therapy for Defining Early Therapy Response in Metastatic Breast Cancer

**DOI:** 10.3390/cancers12041055

**Published:** 2020-04-24

**Authors:** Thomas M. Deutsch, Stefan Stefanovic, Manuel Feisst, Chiara Fischer, Fabian Riedel, Carlo Fremd, Christoph Domschke, Klaus Pantel, Andreas D. Hartkopf, Marc Sutterlin, Sara Y. Brucker, Andreas Schneeweiss, Markus Wallwiener

**Affiliations:** 1 Department of Gynecology and Obstetrics, University Hospital Heidelberg, Im Neuenheimer Feld 440, 69120 Heidelberg, Germany; thomas.deutsch@med.uni-heidelberg.de (T.M.D.);; 2 Department of Gynecology and Obstetrics, Mannheim University Hospital, University of Heidelberg, Theodor-Kutzer-Ufer 1-3, 68167 Mannheim, Germanymarc.suetterlin@umm.de (M.S.); 3 Institute of Medical Biometry and Informatics, University of Heidelberg, Im Neuenheimer Feld 130.3, 69120 Heidelberg, Germany; 4 Department of Medical Oncology, National Center for Tumor Diseases, Im Neuenheimer Feld 460, 69120 Heidelberg, Germany; 5 Institute of Tumor Biology, University Hospital Hamburg-Eppendorf, Martinistrasse 52, 20246 Hamburg, Germany; 6 Department of Gynecology and Obstetrics, University Hospital Tübingen, Calwerstrasse 7, 72076 Tubingen, Germany; andreas.hartkopf@med.uni-tuebingen.de (A.D.H.);; 7 German Cancer Research Center (DKFZ), Im Neuenheimer Feld 280, 69120 Heidelberg, Germany

**Keywords:** breast cancer, circulating tumor cells, cut-off, therapy response, early progression

## Abstract

Detection of circulating tumor cells (CTC) can distinguish between aggressive and indolent metastatic disease in breast cancer patients and is thus considered an independent, negative prognostic factor. A clear decline in CTCs is observed in patients who respond to systemic therapy. Nevertheless, CTCs can decrease in patients experiencing disease progression during systemic therapy, too. This study aims to determine the differences between CTC decline in patients responding to therapy and those in whom disease is progressing. Therefore, CTC values were compared at the start and after one cycle of a new line of systemic therapy. In all, 108 initially CTC-positive patients (with ≥5 intact CTCs in 7.5 mL blood) were enrolled in this study and intact and apoptotic CTCs were measured via the CellSearch^®^ system. A cut-off analysis was performed using Youden’s J statistics to differentiate between CTC change in the two groups. Here, 64 (59.3%) patients showed stable disease or partial response vs. 44 (40.7%) presenting disease progression. Median overall survival was 23 (range: 4–92) vs. 7 (2–43) months (*p* < 0.001). Median intact CTC count at enrollment was 15.0 (5–2760) vs. 30.5 (5–200000) cells (*p* = 0.39) and 2.5 (0–420) vs. 8.5 (0–15000) cells after one cycle of systemic therapy (*p* = 0.001). Median apoptotic CTC count at enrollment was 10.5 (0–1500) vs. 9 (0–800) cells (*p* = 0.475) and 1 (0–200) vs. 3 (0–250) cells after one cycle of systemic therapy (*p* = 0.01). A 50% reduction in baseline apoptotic CTC count represents the optimal cut-off to differentiate between therapy response and disease progression. An apoptotic CTC reduction of ≤10% is 74% specific for early disease progression.

## 1. Introduction

Significant strides have been made in recent years, but metastatic breast cancer (MBC) is still associated with a poor prognosis [1,2,3]. Therefore, identifying patients with the highest risk for disease progression despite current systemic therapy constitutes a challenge that remains vitally important. The presence of circulating tumor cells (CTCs) in the peripheral blood prior to treatment was found to be an independent poor prognostic factor in MBC patients [4,5,6,7,8,9]. Indeed, CTCs can guide the therapy approach by distinguishing between aggressive and indolent metastatic disease [10]. Furthermore, they can be used to monitor treatment response [11,12,13] and have even shown greater prognostic utility than imaging in a single study [14]. Finally, the phenotypic characteristics of CTCs are different from those of the primary tumor and may be predictive for the metastatic tumor phenotype [15,16].

Considering cell morphology, CTCs can be subdivided in intact (iCTCs) and apoptotic (aCTCs) CTCs. Patients with 5 or more iCTCs in 7.5 mL blood are regarded as CTC positive [4,10]. In 52–79% of CTC-positive MBC patients, aCTCs have been observed in peripheral blood samples [17,18,19]. They are thought to be a product of therapy-induced and/or spontaneous apoptosis [20,21]. Whatever the cause of apoptosis may be, significantly higher aCTC counts were detected in patients with MBC than in those with early breast cancer [22]. Janssons et al. revealed that the continuous presence of aCTCs during systemic therapy in MBC was associated with a worse prognosis [23].

The prognostic value of baseline CTC counts and kinetics of CTC number (CTC kinetics) in relation to systemic therapy has been demonstrated in recent studies [6,10,24]. Thus far, either a change in CTC status or a 25% reduction or increase as a cut-off point has been used to define a significant change in CTC counts, rendering them useful for studying the influence of CTC kinetics on breast cancer prognosis [6,7,13,24,25].

The goal of the present study was to assess the utility of the proposed 25% CTC reduction cut-off (iCTC + aCTC, iCTC, aCTC) as a prognostic factor for the lack of disease progression 3 months after initiating of the first cycle of systemic MBC therapy. In addition, we endeavored to identify a more suitable cut-off value.

## 2. Results

The study enrolled 732 patients with MBC who gave their written consent to participate. 107 patients were excluded from the analysis due to missing CTC enumeration at baseline, 229 patients due to missing CTC enumeration after one cycle of systemic therapy, 132 patients due to missing follow-up data, missing clinical data, false or double inclusions, or withdrawal of patients consents for further participation. Finally, 156 patients were excluded because of a negative CTC status at baseline. In total, 108 patients were included in the analysis with a median of 41.0 days (inter quartile range (IQR: 29.0–58.5) between CTC enumeration at baseline and after one cycle of systemic therapy. 64 (59.3%) patients showed stable disease or partial response (SD) and 44 (40.7%) experienced disease progression (PD) at three months (Table 1). The two subgroups (SD and PD) were similar in regard to age at breast cancer diagnosis and at study enrollment, frequency of bone and visceral metastasis, primary tumor hormone receptor expression, and metastatic tumor hormone receptor and HER2 receptor expression (Table 1). However, significant differences in therapeutic modalities were observed between the subgroups, with the patients with PD having been treated with non-first line systemic (chemo-)therapy more often (Table 1).

All patients were iCTC-positive at baseline (as a criterion for inclusion) and the median baseline iCTC counts were similar in patients with SD and PD: 15.0 (range: 5–2760) vs. 30.5 (5–200,000) cells (*p* = 0.39). Furthermore, aCTCs were detected in a similar proportion of patients in both subgroups at baseline (*p* = 0.53) with the median number of detected aCTCs in the subgroups being similar as well (*p* = 0.47).

After one cycle of systemic therapy, 27 patients (42.2%) with SD and 29 patients (65.9%) with PD were iCTC-positive, which represented a statistically significant difference (*p* = 0.02). In addition, the iCTC counts were significantly lower in the SD group than in the PD group: Median 2.5 (0–420) vs. median 8.5 (0–15,000) cells (*p* = 0.001). Similarly, 13 patients (20.3%) with SD and 20 patients (45.5%) with PD were aCTC-positive after one cycle of systemic therapy (*p* = 0.008). Median aCTC counts were also significantly lower in patients with SD than in PD: 1 (0–200) vs. 3 (0–250) cells (*p* = 0.01), respectively.

Although aCTC and iCTC had increased in some patients in both subgroups after therapy (positive values in the ranges presented in rows designated aCTC change and iCTC change in Table 1), significant differences were observed in the median change in aCTC between the subgroups: −6 (−1498–129) vs. 0 (−800–239) (*p* = 0.005) for SD and PD, respectively. In contrast, median iCTC change differed not significantly between SD and PD: −9.5 (−2748–90) vs. −7 (−185000–816) (*p* = 0.172).

**Figure 1:**
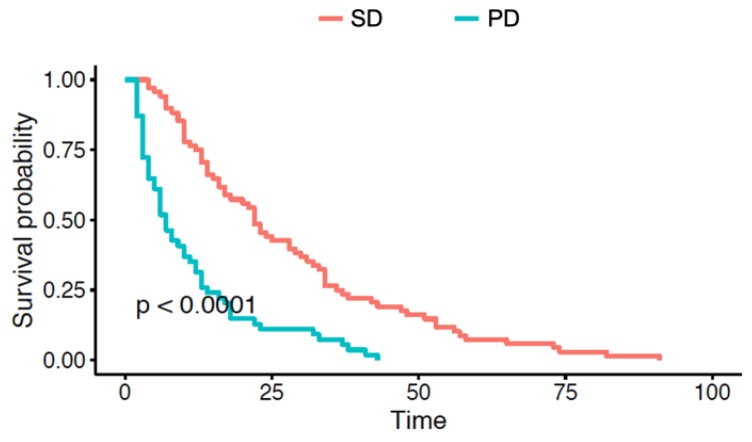
Kaplan–Meier curves representing differences in OS (in months) between patients with early disease progression (PD) and without disease progression (SD) at 3 months after systemic therapy.

Overall survival (OS) was significantly shorter in patients with early PD than in those with early SD: 23 (4–91) vs. 7 (2–43) months and Kaplan–Meier curves were compared using the log-rank test (*p* < 0.001); See Table 1 and Figure 1.

**Figure 2:**
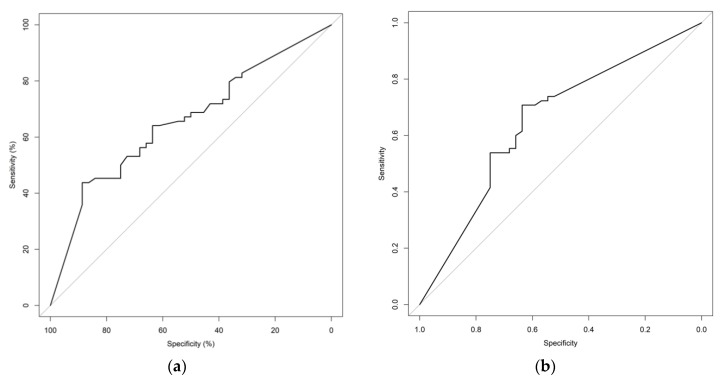

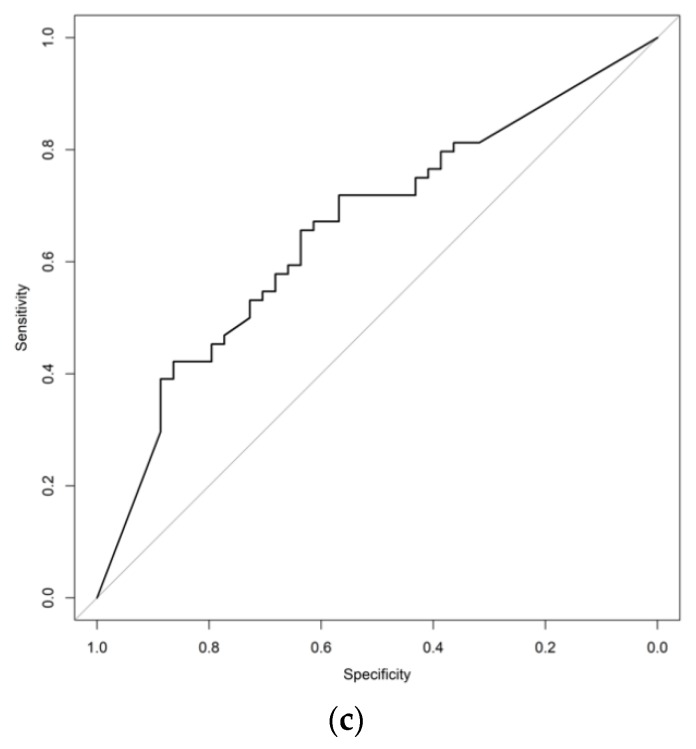
ROC curves representing the sensitivity and specificity of (**a**) relative iCTC reduction (%), (**b**) relative aCTC reduction (%), and (**c**) relative iCTC + aCTC reduction (%) for detecting patients with PD after a single cycle of systemic therapy.

Receiver operating characteristic (ROC) curves illustrating the utility of iCTC, aCTC, and iCTC + aCTC kinetics to identify patients at risk for early disease progression are shown in Figure 2. The iCTC + aCTC ROC had the highest AUC (Table 2). At the 25% reduction cut-off point the sensitivity of the iCTC and iCTC+aCTC was identical at 79.7% while the iCTC had higher specificity at 38.6%. However, the best overall characteristics (best possible combination of sensitivity and specificity) of any single test for detecting of those at risk for early disease progression were a sensitivity of 70.8% and a specificity of 64.6% achieved at a cut-off of a 50% reduction in aCTC counts (Table 2). To calculate the cut-off optimized for sensitivity, a minimum specificity of 50% was set. The best sensitivity (73.9%) was achieved at a cut-off of a 9.8% aCTC reduction.

**Figure 3:**
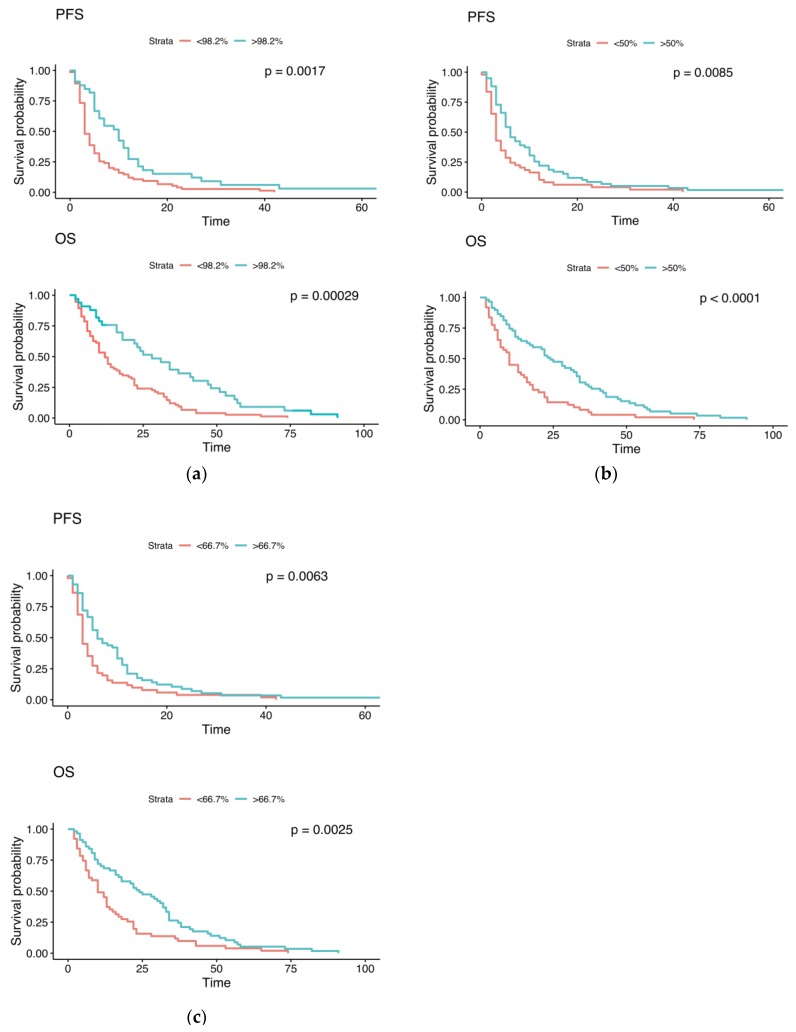
Kaplan–Meier curves representing differences in OS and PFS (in months) between groups with a cut-off of (**a**) a 98.2% iCTC reduction, (**b**) a 50% aCTC reduction, and (**c**) a 66.7% iCTC + aCTC reduction.

The impact of the optimized cut-offs on OS and PFS is shown in Figure 3. Regarding the optimized iCTC cut-off, >98.2% reduction (*n* = 33) vs. <98.2% reduction (*n* = 75) showed a median PFS of 10 (95% confidence interval: 6–12) vs. 3 (3–5) months (*p* = 0.0017) and a median OS of 28 (18–47) vs. 12 (10–17) months (*p* = 0.00029). Regarding the optimized aCTC cut-off, >50% reduction (*n* = 59) vs. <50% reduction (*n* = 49) showed a median PFS of 6 (5–10) vs. 3 (3–5) months (*p* = 0.0085) and a median OS of 24 (17–34) vs. 10 (7–16) months (*p* < 0.0001). Regarding the optimized iCTC+aCTC cut-off, >66.7% reduction (*n* = 57) vs. <66.7% reduction (*n* = 51) showed a median PFS of 6 (5–10) vs. 3 (3–5) months (*p* = 0.0063) and a median OS of 24 [17,18,19,20,21,22,23,24,25,26,27,28,29,30,31,32,33] vs. 10 (7–15) months (*p* = 0.0025).

## 3. Discussion

The negative prognostic value of persisting CTC under ongoing MBC treatment is undisputed and has been proven in multiple studies [6,12,24,26]. Nevertheless, a tendency for CTC to decrease under systemic therapy is evident. However, patients with early progression of disease show a less pronounced decrease in CTC values (Table 1). To distinguish between CTC decline in patients who respond to therapy and those showing early disease progression, optimal cut-off values were defined for the relative decreases of iCTCs, aCTCs, and the combination iCTC + aCTCs.

A 50% reduction in the number of aCTCs after one cycle of systemic therapy was found to be the optimal cut-off for identifying initially CTC-positive patients with a low risk of an early disease progression, which achieved a sensitivity of 70.8% and a specificity of 64.6% with an accuracy of 68.3% (Table 2). Even higher specificity of 88.6% can be reached for a 98.2% decrease of iCTC with an accuracy of 62.0%. Nevertheless, the sensitivity is very low with 43.8%. As an instrument for detection of early non-responders it is therefore not suitable to minimize the risk of unnecessary discontinuation of potentially effective treatments [27]. The Kaplan–Meier plots confirm the impact on PFS and OS for the calculated optimized cut-offs for iCTC, aCTC, and iCTC + aCTC, demonstrating significant differences between the two groups for each cut-off method.

Also interesting is the notion that one could be approximately 74% certain (sensitivity) that early disease progression will occur in a patient if she has not achieved an aCTC reduction of at least 10% from baseline after one cycle of systemic therapy (Table 2). Especially in cases of poor tolerance of a new line of systemic therapy or occurrence of serious adverse events, these findings can help to guide clinicians in critically scrutinizing the chosen therapeutic approach.

Many prognostic factors for MBC have been identified thus far [14,28,29,30,31,32,33]. In a previous study we could show that a 25% therapy-induced reduction in both the number of aCTCs and iCTCs in the peripheral blood were associated with increased survival and progression-free survival (PFS) in the MBC setting [24]. However, the results were less convincing for the iCTC than for the aCTC kinetics. The study arbitrarily used a 25% reduction to define a significant CTC decrease and did not strive to optimize this cut-off.

Two European cohorts analyzed CTC kinetics without considering the absolute or relative change in the number of CTCs—they considered patients in whom CTCs could not be detected after therapy to have experienced a decrease in CTCs [6,34]. Both groups found that stable CTC-positive patients were at highest risk for both disease progression and death. The data also pointed to a better prognosis for patients in whom CTC-status became negative after therapy, but this was less convincing, especially for PFS. Horn et al. demonstrated the prognostic relevance of a significant change value of the CTC count between baseline and three therapy cycles to define a CTC evaluation method for the low and medium risk groups [35]. This method allows to also take initially CTC negative patients into account for CTC decrease. Wang et al. found no significant differences in either OS or PFS comparing their two patients subgroups being treated for MBC—one subgroup included patients who were CTC negative both at baseline and follow-up (3–5 weeks after starting systemic therapy) and patients who had a 50% reduction in the number of CTCs between baseline and follow up, while the other subgroups included all other patients [36]. However, the analysis included a small number of patients and both OS and PFS were longer in the first subgroup; nonetheless, statistical significance was not achieved. A study by Ma et al. evaluated the predictive value of baseline CTC numbers in the non-MBC setting for the success of chemotherapy [37]. The study did not evaluate the prognostic capability of reduced CTC but did provide data on CTC kinetics.

Regarding other cancers, the prognostic role of CTC kinetics under therapy becomes evident. In a combined study for metastatic breast and prostate cancer, Coumans et al. demonstrated that for initially CTC-positive patients, a decrease of CTC count below the cut-off after 6–8 weeks of therapy is the best indicator of treatment response [38]. The association of therapy response and conversion from positive to negative CTC status was also demonstrated for metastatic non-small cell lung cancer, locally advanced head and neck cancer and gastric cancer [39,40,41]. Lorente et al. described a 30% CTC decline 4 weeks after treatment for castration-resistant prostate cancer with initially ≥5 CTCs/7.5 mL as independently associated with OS and a more sensitive biomarker than 50% CTC decline [27]. He could also demonstrate that a percentage decline criterion for response is more sensitive than a conversion from positive to negative CTC status [27].

Our cohort was small, thus limiting the power of the study. A further limitation was that the subgroup in which the patients suffered early disease progression was more often treated with non-first line systemic (chemo-) therapy. This could have led to lower baseline CTC counts due to previous therapies, since CTCs show a general tendency to decrease under systemic therapy. Another limitation of the study was the EpCAM based CellSearch^®^ system which underestimates the count of EpCAM/keratin negative CTCs. Numerous (EpCAM-independent-) CTC enumeration approaches were introduced in the last years, nevertheless, the CellSearch^®^ system is so far the only FDA approved system for CTC enumeration and is the method of choice in the international expert consensus publications [10]. In general, the AUC for all CTC reduction ROC curves was rather small. This might be due to the fact, that the examined cohort had already a very poor prognosis. Regarding only the baseline CTC-positive patients, the study was including only a prognostically unfavorable subpopulation of MBC patients [10]. CTC kinetics in initially CTC-positive patients can therefore provide additional information on the probability for therapy response, but work as a single therapy evaluation instrument. In addition, patients undergoing HER2 targeted therapy have shown substantially decrease in the number of CTCs [42,43]. HER2 positive MBC patients might be underrepresented due to the exclusion of baseline CTC negative patients.

## 4. Materials and Methods

For our prospective, partially blinded cohort study, we enrolled patients treated for MBC at the National Center for Tumor Diseases (NCT), Heidelberg, Germany, and the Department of Obstetrics and Gynecology, University of Heidelberg, Heidelberg, Germany between March 2010 and February 2019. The patients had to have been starting a new line of systemic therapy at study enrollment. Furthermore, peripheral blood samples for enumerating CTCs at baseline and after one cycle of systemic therapy had to have been available. The entire study cohort had to have been reevaluated for disease progression. Only baseline CTC-positive patients (≥5 iCTCs in 7.5 mL blood) were included in this analysis. All subjects have given their informed consent for inclusion before they participated in the study. The study was conducted in accordance with the Declaration of Helsinki, the protocol was approved by the Ethics Committee of the Medical Faculty of the University of Heidelberg, approval no. S-295/2009.

CTCs were enumerated in two 7.5 mL blood samples collected in CellSave tubes (J Janssen Diagnostics, LLC, Raritan, NJ, USA)—one taken prior to treatment and the other after finishing the first cycle of systemic therapy. Samples were kept at room temperature for up to 96 h before analysis using the Cell-Search^®^ assay (CellSearch^®^ Epithelial Cell Kit/CellSpotter^®^ Analyzer, Janssen Diagnostics, LLC, Raritan, NJ, USA) according to the prespecified manufacturer’s instructions. Samples were considered CTC-positive if at least 5 CTCs were identified in the entire sample volume [44]. Morphologically intact, CD45-negative CTCs without any obvious alterations in nuclei and non-speckled keratin immunofluorescence were defined as iCTCs. The aCTCs were visually characterized by speckled keratin staining patterns and/or fragmented or disintegrated nuclei. In addition, some were classified as aCTCs solely by the M30 antibody positivity for the detection of caspase-cleaved Keratin-18 (VLV bio, 1:100) [24]. The majority of aCTCs had characteristic morphologic changes and were also positive for M30.

The patients were assessed for disease progression every 3 months until death or loss to follow-up utilizing the Response Evaluation Criteria in Solid Tumors (RECIST) [45]. The study cohort was divided into two subgroups—patients with stable disease or partial response (SD) and those with early progressive disease (PD) at the first tri-monthly follow-up RECIST evaluation.

ROC curve analyses were used to evaluate the sensitivities and specificities of iCTC and aCTC counts as well as the iCTC + aCTC count for detecting patients without risk of early disease progression. Subsequently, Youden’s J statistics were determined for the three aforementioned ROC curves and the sensitivities and specificities at those points were defined. Finally, we identified sensitivity-optimized cut-off points in an attempt to identify patients who might benefit the most from aggressive treatment while avoiding unnecessarily treating others, thus risking adverse events and increasing treatment costs. Therefore, the lower boundary for specificity was set to be at least 50%, representing even odds.

Treating physicians and patients were unaware of the CTC status of any given patient for the duration of the study. All investigators who performed and/or reviewed the CTC-related measurements and the radiologists who evaluated the patients for signs of disease progression were blinded to patients’ medical histories. Ethical approval was obtained from the Ethics Committee of the Medical Faculty of the University of Heidelberg, approval no. S-295/2009.

Demographic data and clinical characteristics were presented as frequencies and percentages, medians, and ranges, or means and standard deviations as appropriate. Groups were compared using the Wilcoxon rank-sum test or Chi-squared test, as appropriate. Kaplan–Meier plot and the log rank test were utilized for comparing overall survival (OS) and progression free survival (PFS) between the aforementioned patient subgroups. ROC curves were analyzed determining AUC, Youden’s J statistics, and sensitivities and specificities. Statistical analyses were performed using R (version 3.4.1, The R Foundation for Statistical Computing) [46]. A significance level of 5% was chosen. Since this is an exploratory study, *p*-values were not adjusted for multiplicity and have only descriptive meaning.

## 5. Conclusions

We have found that a 50% reduction in baseline aCTC counts after a single cycle of systemic therapy is the optimal cut-off for classifying patients based on risk of early disease progression if they were initially CTC-positive. Furthermore, an aCTC reduction of 10% or less after the first cycle of systemic therapy is 74% specific for early disease progression.

## Figures and Tables

**Table 1:** Patient and tumor characteristics

Statistics	SD	PD	*p*
Total, *n* (%)	64 (100%)	44 (100%)	
iCTC count at baseline, median (range)	15 (5–2760)	30.5 (5–200,000)	0.39
aCTC−positive at baseline, *n* (%)	43 (67.2%)	27 (61.4%)	0.53
aCTC count at baseline, median (range)	10.5 (0–1500)	9 (0–800)	0.475
iCTC−positive after 1 cycle of syst. therapy, *n* (%)	27 (42.2%)	29 (65.9%)	0.02
iCTC count after 1 cycle of syst. therapy, median (range)	2.5 (0–420)	8.5 (0–15,000)	0.001
aCTC−positive after 1 cycle of syst. therapy, *n* (%)	13 (20.3%)	20 (45.5%)	0.005
aCTC count after 1 cycle of syst. therapy, median (range)	1 (0–200)	3 (0–250)	0.01
iCTC change, median (range)	−9.5 (−2748–90)	−7 (−185,000–816)	0.172
aCTC change, median (range)	−6 (−1498–129)	0 (−800–239)	0.005
iCTC + aCTC Baseline	30 (6–4260)	43 (5–200,000)	0.593
iCTC + aCTC after 1 cycle	4 (0–480)	18 (0–15,000)	<0.001
iCTC + aCTC change	1 (−1246–460)	−3 (−185,000–1077)	0.059
Age at initial diagnosis, median (range)	50 (32–81)	48.5 (28–73)	0.07
Age at study enrollment, median (range)	55.5 (36–81)	55.5 (33–77)	0.34
ER−positive primary tumor, *n* (%)	45 (73.8%, NA = 3)	30 (73.2%, NA = 3)	0.95
HER2−positive primary tumor, *n* (%)	14 (23.7%, NA = 5)	4 (11.1%, NA = 8)	0.128
ER−positive metastasis, *n* (%)	27 (81.8%, NA = 11)	19 (73.1%, NA = 18)	0.42
HER2−positive metastasis, *n* (%)	2 (6.3%, NA = 12)	6 (21.4%, NA = 8)	0.08
Number of metastatic sites One site, *n* (%) Multiple sites, *n* (%)	16 (25.0%) 48 (75.0%)	8 (18.2%) 36 (81.8%)	0.49
Site of metastasis Bone, *n* (%) Viscera, *n* (%)	51 (79.7%) 47 (73.4%)	35 (79.5%) 33 (75.0%)	0.99 0.86
Metastatic systemic therapy lines First line, *n* (%) Second line, *n* (%) Other lines of therapy, *n* (%)	13 (17.2%) 28 (43.8%) 25 (39.1%)	5 (11.3%) 13 (29.5%) 26 (59.1%)	0.12
Metastatic chemotherapy lines First line, *n* (%) Second line, *n* (%) Other lines of therapy, *n* (%)	16 (25.0%) 29 (45.3%) 19 (29.7%)	8 (18.2%) 11 (25.0%) 25 (56.8%)	0.02
Median OS in months, median (range)	23 (4–91)	7 (2–43)	<0.001

**Table 2:** ROC characteristics

CTC	Cut-Off	Sensitivity	Specificity	Accuracy	AUC ^1^
iCTC	−25% ^2^	79.7%	36.4%	62.0%	
	J (−98.2%) ^3^	43.8%	88.6%	62.0%	0.657
	OfSn (−58.6%) ^4^	68.8%	50.0%	61.1%	
aCTC	−25% ^2^	72.3%	54.5%	65.0%	
	J (−50%) ^3^	70.8%	64.6%	68.3%	0.651
	OfSn (−9.8%) ^4^	73.9%	54.6%	66.0%	
iCTC + aCTC	−25% ^2^	79.7%	38.6%	63.0%	
	J (−66.7%) ^3^	64.1%	63.6%	63.9%	0.686
	OfSn (−50.3%) ^4^	71.9%	50.0%	63.0%	

^1^ AUC: area under the curve; ^2^ −25%: 25% reduction cut-off; ^3^ J: Youden J point with the percent reduction corresponding to that J point represented in the parentheses; ^4^ OfSn: cut-off optimized for sensitivity with the corresponding percent reduction represented in the parentheses.
